# Enhanced infection and transmission of the 2022–2024 Oropouche virus strain in the North American biting midge *Culicoides sonorensis*

**DOI:** 10.1038/s41598-025-11337-8

**Published:** 2025-07-28

**Authors:** Stacey L.P. Scroggs, Jessica Gutierrez, Lindsey M. Reister-Hendricks, Krista B. Gunter, Natasha L. Tilston, Bethany L. McGregor

**Affiliations:** 1 Arthropod-Borne Animal Diseases Research Unit, Agricultural Research Service, United States Department of Agriculture, Manhattan, KS 66502 USA; 2 Department of Microbiology and Immunology, Indiana University School of Medicine, Indianapolis, IN 46202 USA

**Keywords:** Oropouche virus, *Culicoides sonorensis*, Biting midge, Vector competence, OROV 240023, OROV BeAn19991, Viral vectors, Virology

## Abstract

Oropouche virus (OROV) is a vector-borne zoonotic virus that causes febrile illness in humans. Biting midges of the *Culicoides* genus are the primary vectors during human outbreaks. The 2022–2024 OROV outbreak has seen an increase in incidence, geographic expansion, and the emergence of previously undocumented symptoms. To better understand the basis of increased disease incidence, infection of the outbreak virus (OROV^240023^) was compared to a historical virus strain (rOROV^BeAn19991^) in *Culicoides sonorensis*, a midge species that has demonstrated historical competence. Higher levels of infection, dissemination, and transmission potential were observed in *C. sonorensis* infected with the outbreak strain compared to the historical strain, although infectious titers did not differ between the two viruses. OROV^240023^ was also detected in saliva at earlier time points than rOROV^BeAn19991^, indicating a shorter extrinsic incubation period of < 5 days compared to 7–14 days for rOROV^BeAn19991^. Taken together, our results demonstrate increased transmission potential of the outbreak strain in *C. sonorensis* midges, raising concern about the risk of spread within the United States following potential introduction. However, further studies are needed to evaluate the current strain in *Culicoides* species occurring within its outbreak range, including *Culicoides paraensis*, the confirmed South American vector of OROV.

**Supplementary Information:**

The online version contains supplementary material available at 10.1038/s41598-025-11337-8.

## Introduction

Oropouche virus (OROV, *Peribunyaviridae*, *Orthobunyavirus oropoucheense*) is an emerging arthropod-borne pathogen endemic to Central and South America that causes a febrile illness referred to as Oropouche fever^1,2^. Since its first isolation in 1955 in Trinidad and Tobago from a febrile patient^3^, OROV has caused multiple outbreaks across Brazil, Panama, and Peru, infecting an estimated half a million people. In late 2023, a significant, ongoing, multi-country outbreak was reported in Brazil and to date has spread to 11 countries and one territory, causing over 20,000 confirmed cases, including four deaths^4^. The clinical symptoms of OROV resemble those of other arboviruses like dengue and Zika virus, which likely leads to underreporting of OROV cases. Further, the recent outbreak has revealed that OROV can be vertically transmitted, and infection during pregnancy can result in poor perinatal outcomes, including fetal death and congenital malformations^4,5^.

OROV is zoonotic and is maintained in distinct urban and sylvatic cycles in its historical range. In the urban cycle, OROV is primarily transmitted to humans by *Culicoides* spp. biting midges, a genus of minute flies that are known to transmit numerous viruses, primarily of veterinary importance^1,6–9^. Of particular significance to OROV transmission in its historical range is *Culicoides paraensis*, a species found in both urban and rural environments^10^. This species has been documented breeding extensively in rural cacao and banana plantations, offering an opportunity for the potential bridging of OROV between cycles^10–12^, and is also found inhabiting moist tree-holes and habitats with rotting organic matter present^13,14^. The sylvatic cycle is still relatively poorly understood. OROV is thought to be maintained in this cycle by biting midge species and numerous wildlife reservoir hosts such as sloths and primates^15–18^. Antibody evidence demonstrates that some domestic animals, livestock, and birds can be infected by OROV^19–21^. Further studies are needed to determine if these species are competent reservoir or amplification hosts. *Culex quinquefasciatus*, a species that is often listed as an OROV vector, has shown inefficient transmission of OROV in laboratory assays^22,23^, however this low competence may be overcome by high population sizes in some areas. The role of other mosquito species in OROV transmission is poorly understood.

OROV has a negative-sense, tri-segmented RNA genome that is capable of genetic reassortment^1,24^. This evolutionary process can result in the emergence of new viral strains with epidemic potential, including enhanced virulence and altered pathogenicity^25^. Interspecies reassortment events involving OROV have resulted in the emergence of novel viruses such as Iquitos virus, Madre de Dios virus, and Perdões virus, which currently circulate in South America at unknown frequencies^26,27^. The novel 2022–2024 OROV strain, referred to here as OROV^240023^, is believed to be a reassortant derived from diverse circulating OROV S, M, and L lineages^25,26,28,29^. Emerging evidence suggests that this strain may have increased replication competence and distinct disease phenotypes^25^. Experimental data also indicate that OROV^240023^ exhibits partial escape from neutralization antibodies generated against previous OROV exposures^25,30^.

The ongoing OROV epidemic is a threat to public health in the United States. As of May 2025, a total of 109 travel-associated cases have been confirmed, two of which were neuroinvasive, in seven states and territories, primarily Florida^31^. Local transmission has not yet been documented, but the presence of travel-associated cases co-located with competent vectors and a naive host population increases the risk of OROV emergence and establishment. To understand the risk of OROV emergence into North America, the infection, dissemination, and transmission risk of North American arthropod vectors must be evaluated. Laboratory infections of *Culicoides sonorensis*, a biting midge species distributed widely across Mexico and the United States^32^, with the historic strain of OROV isolated in 1955, demonstrated high infection and dissemination rates (> 80% of bodies and heads positive for OROV) and moderate transmission potential (19% of salivary samples positive for OROV)^22^. Based on these findings and the increase of OROV^240023^ fitness in mammalian cells^25^, we hypothesized that OROV^240023^ would exhibit a fitness advantage over a historical OROV isolate in *C. sonorensis* biting midges. If OROV^240023^ is more fit in biting midges, then the risk of OROV establishing a local transmission cycle within the United States is elevated. Understanding the transmission potential of contemporary OROV strains in local vector species is imperative for designing effective public health and vector control policies to prevent and mitigate future outbreaks.

## Results

### OROV^240023^ replicates to higher levels in midge cells compared to rOROV^BeAn19991^

**Fig. 1:**
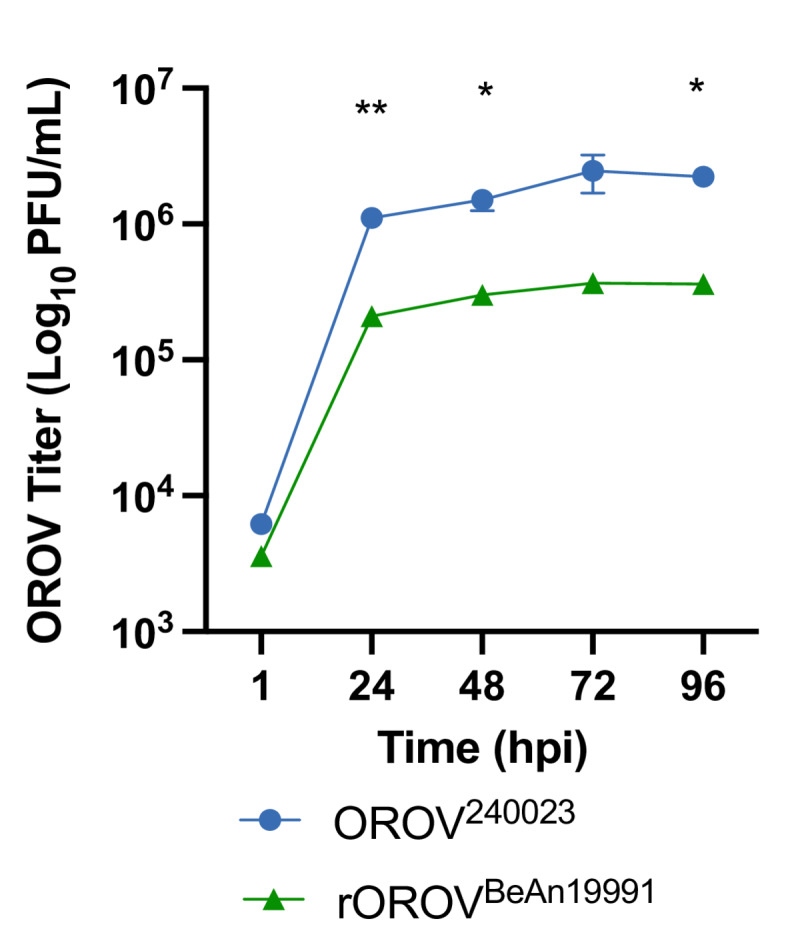
Replication kinetics of OROV^240023^ (blue circles) and rOROV^BeAn19991^ (green triangles) in W8 midge cells. Average viral titer (+/- SE) indicated. Tukey pairwise comparisons * p<0.05, ** p<0.01. hpi, hours post infection.

Multicycle replication curves of OROV^240023^ and rOROV^BeAn19991^ in midge cells reveal that the outbreak isolate OROV^240023^ replicates to higher viral titers 24-, 48-, and 96-hours post-infection (HPI) compared to the historical strain rOROV^BeAn19991^ (Fig. 1, Repeated Measures Two-Way ANOVA F (4,16) = 5.8, *p* = 0.004). After 24 HPI, the viral titers for OROV^240023^ were on average 5.8-fold higher than the viral titers of rOROV^BeAn19991^.

### Sequence identity between OROV^240023^ and rOROV^BeAn19991^ depends on the segment

Segment-specific alignments of OROV^240023^ and rOROV^BeAn19991^ demonstrate that the percent pairwise identity of the two viruses varies by segment. Segments S and M exhibit the highest nucleotide and amino acid pairwise identities (Table 1). As segment S encodes two proteins from overlapping regions of the same mRNA^33^, separate amino acid percent identities are reported for the nucleocapsid (N) and nonstructural protein (NSs). The percent identities from the L segment, which contains the RNA dependent RNA polymerase, are the lowest of the three segments (Table 1).

**Table 1:** Nucleotide and amino acid percent identities for OROV^240023^ and rOROV^BeAn19991^.

Segment	Nucleotide % Identity	Amino Acid % Identity
S	95.4	N: 100; NSs: 97.8
M	96.2	98.2
L	89.6	96.9

### OROV^240023^ was detected in more pools of midges compared to rOROV^BeAn19991^, but viral titers were equivalent

**Fig. 2:**
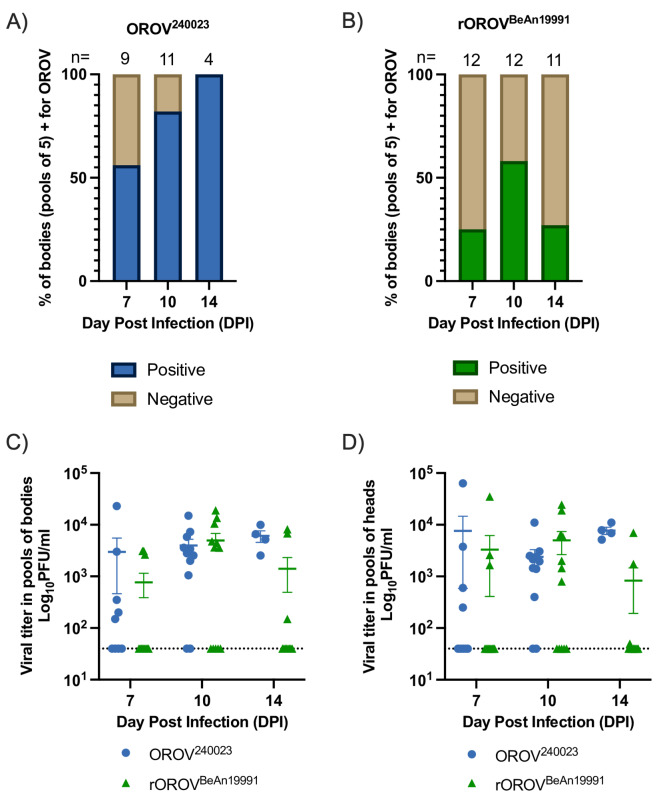
OROV^240023^and rOROV^BeAn19991^in live midges. Percent of midge body pools positive for infectious virus after infection with OROV^240023^ (**A**) or rOROV^BeAn19991^(**B**). Number of pools (n = 5 midge bodies per pool) collected for each virus at each time point indicated above each bar. **C**) Viral titers for pools of 5 midge bodies infected with OROV^240023^(blue circles) or rOROV^BeAn19991^(green triangles). **D**) Viral titers for pools of 5 midge heads infected with OROV^240023^(blue circles) or rOROV^BeAn19991^(green triangles). Average viral titers (+/- SE) and the limit of detection (dashed line) indicated.

After feeding on the spiked bloodmeals for one hour, 10 individual, fully blood-fed females were collected for both viruses, and all 20 tested positive for OROV. The average whole-body titers were 4.55 log_10_PFU/mL for the OROV^240023^group and 4.62 log_10_PFU/mL for rOROV^BeAn19991^. After incubation, most midge body pools that fed on the OROV^240034^-spiked infectious bloodmeal tested positive for infectious virus (Fig. 2A). By 7 days post-infection (DPI), 56% of the body pools (*n* = 9 total pools) were positive for virus, which increased to 82% (*n* = 11 total pools) on 10 DPI, and 100% on 14 DPI, although it should be noted only 4 pools were collected 14 DPI. Fewer midge body pools that fed on the rOROV^BeAn19991^-spiked infectious bloodmeal tested positive (Fig. 2B). On 7 DPI, 25% of the body pools (*n* = 12 total pools) were positive. The maximum amount of rOROV^BeAn19991^ positive body pools (58%) was detected 10 DPI (*n* = 12 total pools). By 14 DPI, the number of positive body pools had dropped significantly to 3 out of 11 (27%). The difference in number of positive pools by virus and time point was significant (χ^2^ = 12.5, df = 1, *p* = 0.0004). The mean viral titers for the midge body pools did not differ by virus or by time point (Fig. 2C; Mixed effect model p-value > 0.05) and ranged between 3.1 and 3.7 log_10_PFU/mL.

All pools of midge heads that correspond to pools of OROV-positive bodies were also OROV positive, but the mean viral titers did not differ by virus or time point (Fig. 2D; Mixed effect model p-value > 0.05). The mean viral titers of body pools vs. head pools by virus were not different (Mixed effect model p-value > 0.05).

The OROV^240023^ pre- and post-bloodmeal viral titers were 6.77 log_10_ PFU/mL and 6.67 log_10_ PFU/mL. The rOROV^BeAn19991^ pre- and post-bloodmeal viral titers were 6.85 log_10_ PFU/mL and 6.74 log_10_ PFU/mL.

### Detection of OROV^240023^ and rOROV^BeAn19991^ in individual midges was low compared to pools

Heads and bodies from twenty individual midges per virus and timepoint (7-, 10-, and 14 DPI) were collected. Infectious virus was detected in individual midge bodies and heads, but the number of positive midges was low for both viruses at all time points. Most OROV-positive midges were collected at 14 DPI. For both viruses, four out of 20 bodies (20%) and only one corresponding head were positive. The average viral titers were 2.85 log_10_ PFU/mL for OROV^240023^ and 2.39 log_10_ PFU/mL for rOROV^BeAn19991^. Only one midge body per virus (5%) was positive from 10 DPI. Of those two heads, only the head of the midge infected with OROV^240023^ was positive. None of the 7 DPI midges infected with OROV^240023^ were positive, but 1 (5%) from the rOROV^BeAn19991^-infected midges was positive (body and head).

### OROV^240023^ was detected earlier and more frequently in saliva from pooled midges compared to rOROV^BeAn19991^

**Fig. 3:**
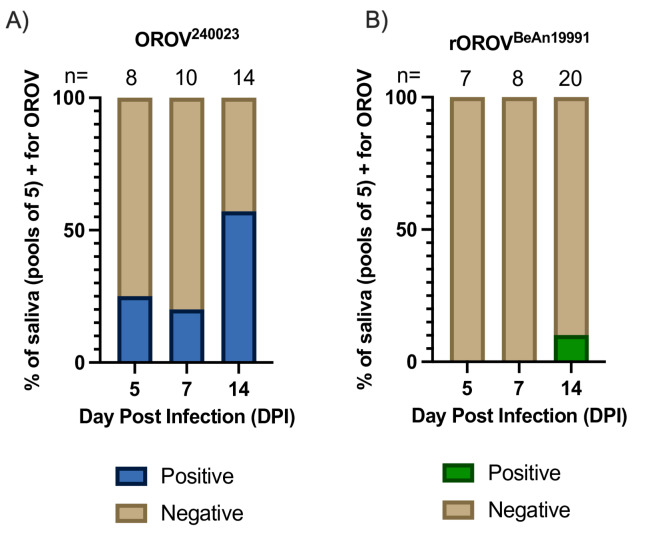
OROV^240023^and rOROV^BeAn19991^in midge saliva. Percent of midge saliva pools positive for OROV after midge infection with OROV^240023^ (**A**) or rOROV^BeAn19991^ (**B**). Number of pools (n = 5 midge bodies per pool) collected for each virus at each time point indicated above each bar.

OROV^240023^ was detected in more pools of saliva via cytopathic effect (CPE) and confirmed by RT-qPCR on DPI 5 (25% of 8 saliva pools), 7 (20% of 10 saliva pools), and 14 (58% of 14 saliva pools), while rOROV^BeAn19991^ was only detected in saliva from midges on 14 DPI (10% of 20 saliva pools) (Fig. 3A and B; Fisher’s Exact p-value = 0.03).

## Discussion

OROV is an emerging zoonotic arbovirus that regularly initiates outbreaks in South America. The primary vector species of OROV is *Culicoides paraensis*, whose geographic distribution extends from Brazil and Argentina up to Wisconsin in the United States^34^, but there are over 1300 species of *Culicoides* biting midges worldwide, of which about 30 are known or suspected vectors of viruses^35^. In the United States there are about 150 species of biting midges^34^, including *C. sonorensis*, which is a vector of the agriculturally important arboviruses, bluetongue virus, epizootic hemorrhagic disease virus, and vesicular stomatitis virus^32^. The presence of both travel-associated cases and susceptible biting midge species raises concerns about local OROV transmission and endemicity. *Culicoides sonorensis* has been experimentally demonstrated to be a competent vector for OROV using a historical isolate of OROV originally isolated in 1955 from a febrile patient (OROV TRVL 9760) and a colony of midges (Ausman) that has been maintained in the lab for 24 years^22^. The current study evaluated the risk of infection, dissemination, and transmission potential of the 2024 outbreak OROV strain in a colony of *C. sonorensis* established 5 years ago.

More OROV-positive pools were detected at earlier time points post-infection for midges infected with the circulating OROV isolate OROV^240023^ compared to the lab strain rOROV^BeAn19991^. However, the viral titers of positive pools, regardless of tissue type, did not differ. OROV was similarly detected earlier and in more pools of saliva from midges infected with OROV^240023^. Together, these results suggest that OROV^240023^ infects and replicates more efficiently in the newly established Kansas colony of *C. sonorensis* biting midges, which could decrease the extrinsic incubation period (EIP) and enhance viral transmission of the outbreak virus. Based on pooled saliva samples, we estimate the EIP to be less than 5 days for OROV^240023^ and between 7 and 14 days for rOROV^BeAn19991^. The shortened EIP has major implications for disease transmission and could explain the widespread and ongoing transmission of OROV^240023^. However, these data are from *C. sonorensis* not *C. paraensis*, the assumed primary vector of the outbreak. Determining the EIP of OROV^240023^ in *C. paraensis* is paramount for understanding the ongoing outbreak. These data do suggest an increased risk of viral emergence and establishment in the United States should local populations of *C. sonorensis* become infected with OROV isolates from the current outbreak. Infectious OROV was more readily detected in pools of 5 compared to the individual midge bodies, heads, or saliva. Viral infection of midges depends on the viremia of their bloodmeal. Pinheiro et al., fed *C. paraensis* midges on human patients with varying viremia levels and found 13% of midges became infected with OROV when viremia was between 5.3 and 6.2 log_10_ SMLD_50_ (suckling mouse lethal dose) but midge infection rose to 34% when the viremia was between 6.3 and 7.3 log_10_ SMLD_50_^36^. However, midge species could impact the infectious dose required for midge infection. McGregor et al., fed *C. sonorensis* midges bloodmeals with 6.5 log_10_PFU/mL OROV TRVL 9760 and achieved 87% infection 14 DPI in individual midges^22^. The current study started with 7 log_10_ PFU/mL OROV, and the viral titer dropped one log during the feeding. The infection resulted in 100% infection for midges fed OROV^240023^ and 27% infection for midges fed rOROV^BeAn19991^ by 14 DPI. These two studies varied in the midge colonies (Ausman vs. Kansas) and historical OROV isolate (TRVL 9760 vs. BeAn^19991^), which could explain the differences in percentage of infection on 14 DPI. Our findings are primarily based on data from pools rather than singleton midges, so the results should not be directly extrapolated to individual midges.

The observed discrepancy in infection, dissemination, and transmission potential between the strains in this study suggests the presence of variation in vector competence. The Kansas *C. sonorensis* colony demonstrated a greater midgut infection and escape barrier, and salivary gland infection and escape barrier to the historical rOROV^BeAn19991^ virus. The McGregor et al., 2021, study investigating the OROV TRVL 9760 strain in the Ausman colony of *C. sonorensis* determined that a midgut infection barrier was lacking but demonstrated a moderate salivary gland infection and escape barrier^22^. While the specific mechanisms contributing to this variability are still unknown for most midge-borne pathogens, several factors are believed to contribute to variability in vector competence between individual midges, populations, and viral strains^37^. These results emphasize the need to investigate strain differences in relevant vector populations.

Scachetti et al., 2024, reported that viremia from patients infected with the 2022–2024 circulating OROV was higher than infection with other OROV isolates^38^. Viral replication in two human cell lines and one monkey cell line was also greater than that of the historical isolate OROV^BeAn19991^^38^. In the current study, we found that OROV^240023^ replication in a midge cell line is also enhanced compared to the historical rOROV^BeAn19991^. The OROV^240023^ L segment contained the most amino acid substitutions (*n* = 69) compared to rOROV^BeAn19991^. Combined, these data suggest an overall increase in viral fitness for the 2022–2024 OROV isolates, possibly due to mutations in the RNA-dependent RNA polymerase.

There are important caveats to consider in interpreting the presented results. One such caveat is the temperature-sensitive nature of viruses and vectors^39–43^. The experiments reported in this study were conducted at the same constant temperature (28 °C for cell infections and 25 °C for midge infections). Further studies are needed to determine the impact that temperature has on strain differences and any implications for replication dynamics, dissemination, and transmission. Additionally, human behavior and socioeconomic status play a role in the risk of virus transmission and should be considered when developing effective vector control strategies in the United States to limit *Culicoides*-transmitted viruses^44,45^.

Considering the ability of OROV^240023^ to replicate more efficiently in vertebrate hosts and *Culicoides* biting midges, it is not surprising that the world experienced the largest documented outbreak of Oropouche fever to date. However, there are still many critical gaps in our understanding of OROV that prevent us from responding effectively to current and future outbreaks. Laboratory experiments, along with field surveillance, are essential for understanding competent OROV vectors, which will support the development and execution of effective control strategies.

## Methods

### Cells, viruses, and midges

African green monkey kidney Vero MARU cells (Middle America Research Unit, Panama) were grown at 37 °C with 5% CO_2_ in 199E media supplemented with 2% fetal bovine serum (FBS), 100U penicillin/streptomycin sulfate, and 0.25 µg/mL of amphotericin B. *Culicoides sonorensis* W8 cells, derived from 1-day-old embryonated eggs (USDA ARS Arthropod-Borne Animal Diseases Research Unit, Manhattan, KS, USA) were grown at 28 °C in Schneider’s insect media (MilliporeSigma, St. Louis, MO, USA) supplemented with 0.4 g/L sodium bicarbonate, 18 µL of 10 mg/L bovine insulin, and 5% FBS^46^.

Two OROV isolates were utilized for this study, a historic isolate (strain BeAn19991) and the 2024 outbreak isolate (strain 240023). We used a recombinant version of strain BeAn19991 (rOROV^BeAn19991^), previously described^47,48^. OROV strain BeAn19991 was originally isolated in 1960 from a pale-throated sloth (*B. tridactylus*) and passaged three times in Vero cells. OROV strain 240023 (OROV^240023^) was kindly provided by the Centers for Disease Control and Prevention after isolation in 2024 from a travel-associated case of OROV acquired in Cuba and diagnosed in Florida, then passaged three times in Vero cells. Both stocks were concentrated via ultracentrifugation and stored at −80 °C. Segment-specific alignments for rOROV^BeAn19991^ (accession # NC_005775) and OROV^240023^ (accession # PQ417948) were generated using Geneious Prime^®^ (version 2025.0.2).

*Culicoides sonorensis* biting midges used for this study were from the Kansas Colony, which was colonized in 2020 from a wild population collected from Riley County, Kansas, USA. This colony is maintained at the Center for Grain and Animal Health Research in Manhattan, KS, USA. Adult midges were maintained at 25 °C ±1 °C and 75% relative humidity in environmental chambers set to a 13:11 light: dark cycle. The midges were offered 10% sucrose *ad libitum*.

### Multi-cycle OROV replication curve in midge cells

Triplicate T25 flasks of W8 cells per virus were infected at MOI 0.1 with rOROV^BeAn19991^ or OROV^240023^. After a 2 h incubation at 28 °C with gentle rocking every 20 min, the inoculum was removed by pipette and each flask was washed twice with 1 mL 1x phosphate buffered saline (PBS). The second wash was collected from each flask for the 0 h post-infection (HPI) time point. After the washes, 5 ml of W8 media was added to each flask and the flasks were then incubated at 28 °C. Viral supernatants were collected 24, 48, 72, and 96 HPI. At each timepoint, 1 ml of media from each flask was removed, clarified by centrifugation at 1200 rpm for 10 min at 4 °C, then stored at −80 °C. After media removal, 1 ml of fresh media was added back to each flask.

### Time course of OROV infection in *Culicoides sonorensis* midges

Twenty-four hours prior to infection, midges were provided with water only to encourage blood feeding. Defibrinated sheep blood (Lampire Biological Products, Pipersville, PA, USA) was mixed 1:1 with equal titers of rOROV^BeAn19991^ or OROV^240023^ (7 log_10_ PFU/mL) to make two infectious blood meals. Blood was also mixed 1:1 with cell culture media to make an uninfected control bloodmeal. Midges were allowed to feed using an artificial membrane feeding system for 1 h then anesthetized with CO_2_. Fully engorged, blood-fed females were sorted into cardboard cages with moist egg cups^49,50^. Males and unfed females were discarded. Ten fully engorged, blood-fed midges per virus were collected into individual tubes with 1.4 mm ceramic beads (Omni International, Kennesaw, GA, USA) and 250 µl antibiotic media (M199E media with 2% FBS, 400 µg/mL streptomycin, 400 U/mL penicillin, 200 µg/mL gentamycin, 25 µg/mL ciprofloxacin, and 5 µg/mL fungizone) and immediately stored at −80 °C as feeding controls. Additionally, 100 µl of each infectious bloodmeal was collected into 900 µl Hanks’ Balanced Salt Solution (400 mg/L KCl, 60 mg/L NH_2_PO_4_, 8000 mg/L NaCl, 350 mg/L NaHCO_3_, 48 mg/L Na_2_HPO_4_, and 1000 mg/L D-Glucose) at the beginning (pre-bloodmeal) and at the end (post-bloodmeal) of the 1 h feeding time then stored at −80 °C.

Midges were collected at three time points, 7-, 10-, and 14-days post infection (DPI). At the time of collection, the number of dead and alive were totaled and the live midges were pooled into groups of 5 on a fly pad connected to CO_2_. Heads were removed from bodies. Pools of heads and pools of bodies were placed into separate tubes with 500 µL antibiotic media and 1.4 mm ceramic beads then stored at −80 °C. Individual heads and bodies were also collected into separate tubes with 250 µl antibiotic media with 1.4 mm ceramic beads then stored at −80 °C.

### Virus quantification via plaque assay

Infectious OROV was quantified via plaque assay. Vero MARU cells were seeded in 24-well plates at 1 × 10^6^ cells per well two days before the infection. Pools of midge heads and bodies or individual midge heads and bodies were thawed on ice and homogenized using the Bead Ruptor Elite (Omni International, Kennesaw, GA, USA) for 1 min (two 30 s cycles with a 10 s dwell) at 2.9 m/s before centrifugation at 12,000xg for 8 min at 4 °C to pellet tissue debris. Samples were diluted ten-fold in cell culture media and inoculated with 100 µL onto cell monolayers in duplicate. Plates were rocked for 2 h at 37 °C before overlaid with 1% methylcellulose (4000 cp.) in supplemented MEM media. Plates were incubated at 37 °C for 4 d then fixed and stained with crystal violet formaldehyde to visualize and count plaques.

### Salivary assay with OROV-infected *Culicoides sonorensis* midges

Midges were fed infectious bloodmeals spiked with rOROV^BeAn19991^ or OROV^240023^ and maintained for 5, 7, or 14 DPI as described above, except the ratio of blood to virus was 1:2. Capillary assay was conducted as previously described^22^. On specified DPI, midges were immobilized on tape and their legs and wings were removed. A capillary tube soaked in immersion oil was placed over their mouthparts. After 1 h, the capillary tube was moved to a tube containing 100 µL of media in groups of 5 and stored at −80 °C. The midge legs, wings, bodies, and heads were also collected and stored at −80 °C.

Due to low levels of detection in saliva, virus was quantified by cytopathic effect (CPE) in 96 well plates of Vero MARU cells rather than plaque assay. After centrifugation for 8 mins at 12,000xg, the capillary tubes were removed and 50µL of sample was inoculated onto the confluent cells. After 1 hour, 150 µL of media was added to each well and the cells were incubated at 37 °C and checked daily for signs of CPE. After 5 days, supernatants were collected and RNA was extracted using the MagMAX ^TM^ CORE Nucleic Acid Purification Kit (Applied Biosystems; ThermoFisher Scientific, Inc., Waltham, MA, USA) with the KingFisher X ^TM^ Apex System (Applied Biosystems; Thermo Scientific, Inc., Waltham, MA, USA) according to the manufacturer’s protocol. Virus-induced CPE was confirmed by RT-qPCR using the TaqMan Fast Virus 1-step Master Mix (Applied Biosystems; ThermoFisher Scientific, Inc., Waltham, MA, USA) targeting the S segment with the following primers: forward 5’ TCCGGAGGCAGCATATGTG 3’, reverse, 5’ ACAACACCAGCATTGAGCACTTT 3’ and probe 5’(FAM) CATTTGAAGCTAGATACGGG 3’^51^ using the fast cycling mode described in the manufacturer’s protocol. All reactions were conducted in duplicate and included a 10-fold dilution standard curve using extracted rOROV^BeAn19991^ RNA and a water negative control. Ct values ≤ 36 were considered positive.

### Statistical analyses

Repeated measures two-way ANOVA with Tukey pairwise comparisons were used to analyze viral titer data taken on multiple time points from multicycle replication curve and midge infections. Mixed-effects models were used to analyze differences in viral titers from midge bodies collected at multiple time points. Percent of pools positive or percent of individual midges positive for OROV by time point was analyzed with contingency tables reporting χ^2^ tests statistics. Statistics were conducted using Prism (v.10.4.1). The salivary assay was analyzed with a Fisher’s Exact test in RStudio (v. 2024.09.1 + 394).

## Electronic supplementary material

Below is the link to the electronic supplementary material.

Supplementary Material 1

## Data Availability

All data generated during this study are included in this published article and its Supplemental Information files.
