# Supplementary material for: Enhanced infection and transmission of the 2022–2024 Oropouche virus strain in the North American biting midge Culicoides sonorensis
Source: Sci Rep. 2025 Jul 28;15:27368. doi: 10.1038/s41598-025-11337-8 (PMC12301478; doi:10.1038/s41598-025-11337-8)
Supplement: Supplementary file 1 — Supplementary Material 1 [file 41598_2025_11337_MOESM1_ESM.pdf]

Table S1. Average viral titers for $OROV^{240023}$ and $rOROV^{BeAn19991}$ in W8 cells (Figure 1)

	Average Viral Titer, Log_{10} PFU/mL (+/- SE)	
Time post infection (hours)	$OROV^{240023}$	$rOROV^{BeAn19991}$
1	3.96 (0.03)	3.56 (0.02)
24	6.04 (0.03)	5.32 (0.01)
48	6.17 (0.07)	5.48 (0.04)
72	6.33 (0.17)	5.57 (0.02)
96	6.34 (0.06)	5.55 (0.07)

Table S2. Average viral titers for $OROV^{240023}$ and $rOROV^{BeAn19991}$ in pools of midges by tissue type (Figures 2C, 2D)

		Average Viral Titer, Log_{10} PFU/mL (+/- SE)	
Time post infection (days)	Tissue	$OROV^{240023}$	$rOROV^{BeAn19991}$
0	Whole Body	4.55 (0.07)	4.62 (0.08)
7	Body	2.97 (0.31)	3.47 (0.02)
	Head	3.39 (0.53)	3.73 (0.41)
10	Body	3.56 (0.11)	3.84 (0.11)
	Head	3.32 (0.13)	3.66 (0.21)
14	Body	3.74 (0.12)	3.31 (0.57)
	Head	3.87 (0.07)	2.93 (0.64)
